# Mapping hard tissue maturation: the role of Gallego stain in differential staining

**DOI:** 10.1016/j.jobcr.2025.06.004

**Published:** 2025-06-12

**Authors:** Shweta Mary Reddy, Suganya Panneer Selvam, Ramya Ramadoss, Sandhya Sundar

**Affiliations:** Department of Oral Biology, Saveetha Dental College and Hospital, Saveetha Institute of Medical and Technical Sciences, Chennai, 600077, India

**Keywords:** Bone, Cartilage, Collagen, Dentin, Enamel, Tooth germ

## Abstract

Histological stains are specific dyes that attach to different tissues and are used in conjunction with hematoxylin and eosin stains in routine histopathology to provide useful information on tissues. With the usual stains, it becomes difficult to distinguish between the different hard tissues present in oral pathological disorders. As one of the differential stains to discriminate between the hard tissues, modified Gallego's stain can be utilized. Maxilla and mandible of a 3-day-old rabbit are collected, followed by processing and embedding of the tissues, thin paraffin sections were made & stained with Modified Gallego's, and then viewed under the microscope. In the developing tooth germ, Dentin is stained green, and enamel is stained red in Modified Gallego staining. Modified Gallego staining facilitated the identification of the characteristics of hard and soft tissues during development and the changes in their behavior during maturation.

## 1 Introduction

Tooth development is a multifaceted process characterized by distinct stages such as bud, cap, early bell, and late bell stages. During this process, cells undergo sequential proliferation and differentiation to form teeth and their surrounding structures. Most research on odontogenesis has been carried out in mice, where tooth development is classified into the placode, bud, cap, bell, and maturation stages. In rabbits, the dentition is diphyodont, consisting of two upper and one lower incisor, three upper and two lower premolars, and three upper and three lower molars on each side.^1^^,^^2^ In light of this, our study aimed to investigate the patterning and mineralization of the rabbit tooth germ and the surrounding bone.

Histological staining is a multi-step technical process that involves fixation, processing, embedding, sectioning, and staining using chemical and molecular biology assays.^3^ Histochemistry is a technique that enables an understanding of the chemical composition of cells and tissues, providing insight into the embryonic nature of the tissues. Special stains serve as rapid detectives, identifying the nature of hard tissues and delineating cells in soft tissues, especially in pathology. While several special stains have been employed to study mineralized tissues, literature on their application during *odontogenesis* remains scarce. Most existing studies focus on adult or pathological specimens, leaving a significant gap in understanding the histochemical differentiation and mineralization patterns during early tooth development. The Modified Gallego stain, a variation of Lille's stain that utilizes hematoxylin, carbol fuchsin, and aniline blue, is known for its ability to differentiate bone from cementum-like tissues.^4^ However, its utility in identifying mineralized components in developing dental tissues has not been systematically explored. The Modified Gallego stain was chosen for this study due to its enhanced differential staining capability, particularly in distinguishing bone from cementum-like tissues, which is crucial when analyzing mineralized structures during tooth development. While Lille's stain serves as the base for this technique, the modification enhances contrast and specificity, especially in tissues where mineralization is still in progress or where overlapping characteristics of bone and cementum can make differentiation challenging. This makes it highly advantageous when compared to other conventional stains that may not provide the same level of contrast or specificity for mineralized tissues in early developmental stages.

This study aims to evaluate the effectiveness of the Modified Gallego stain in identifying and differentiating mineralized components of dental tissues and surrounding bone during the early stages of tooth development in rabbit tooth germs. Specifically, the study seeks to apply the Modified Gallego staining technique to histological sections of developing rabbit tooth germs and assess the staining patterns of dentin, cementum, and alveolar bone. By doing so, the study aims to determine the level of visual clarity and contrast this stain provides compared to conventional methods. Additionally, the study explores the potential of the Modified Gallego stain as a valuable tool in developmental dental histology and histopathology, particularly in the context of understanding tissue patterning and mineralization during odontogenesis. Articles on special stains during odontogenesis are limited. Therefore, this study is focused on the patterning and mineralization of dental tissues and surrounding bone in the rabbit tooth germ using the Modified Gallego stain.

## 2 Materials and methods

This study includes the maxilla & mandible from five fetal rabbits. The study protocol was duly approved by the institutional ethical committee (BRULAC/SDCH/SIMATS/IAEC/04–2022/105). The samples were fixed in 10 % neutral buffered formalin and decalcified in 10 % hydrochloric acid for an hour. Later, the samples were placed in increasing grades of alcohol (70 %, 90 %, and 100 %) for 1 h each. The tissue samples were then subjected to xylene (clearing agent) for 15 min, followed by wax impregnation for an hour and made into paraffin blocks. The blocks were then cut into thin sections using a microtome. The slides along with the tissue samples were stained using the routine hematoxylin and eosin, and Modified Gallego stain technique.

### 2.1 Hematoxylin and eosin staining procedure

The tissue section slides are immersed in xylene for 10 min, then hydrated through a series of graded alcohols (70 %, 80 %, 90 % and 100 %) and subsequently rinsed in tap water. Subsequently, the tissue is immersed in Harris hematoxylin stain for 8 min, rinsed in tap water, and finally dipped in ethyl alcohol before being washed in running water. As a final step, the tissue is immersed in eosin for a minute and washed with water. After drying, a cover slip is placed on the slide.

### 2.2 Modified Gallego's staining procedure

**Fig. 1:**
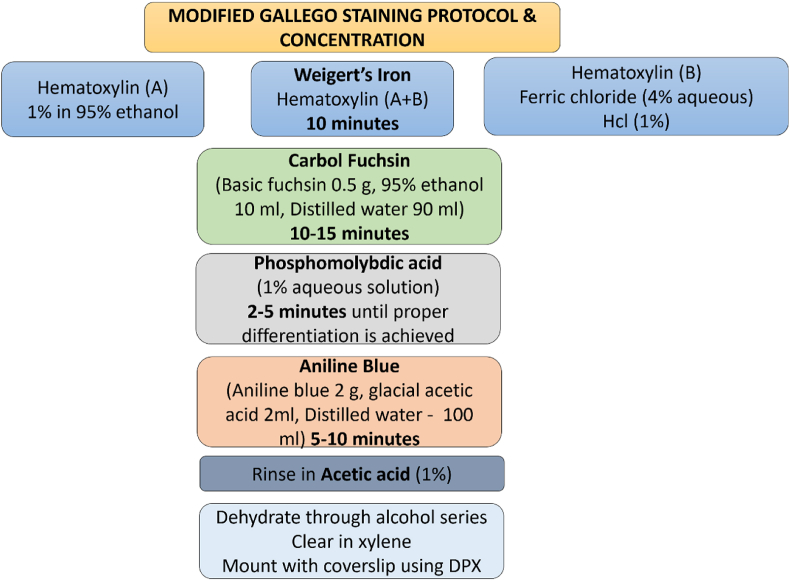
exhibiting the protocol of Modified Gallego stain and concentration of each reagent used (Hcl – Hydrochloric acid, g – gram, ml – milliliter, DPX - Distrene Plasticizer Xylene).

The original Gallego stain utilizes acid fuchsin and picric acid for staining connective tissues. At the same time, the modified version incorporates basic fuchsin, iron alum, and phosphomolybdic acid to enhance tissue differentiation, particularly in hard tissues like teeth. The sections of the tooth germ were subjected to the Modified Gallego staining (0.5 % Aqueous Basic Fuchsin, 4 % Aqueous Iron Alum, 1 % Aqueous Phosphomolybdic Acid) protocol for 15 min, followed by immersion in xylene for 10 min.^5^ The sample was then rinsed with tap water and subsequently subjected to hematoxylin staining. The tissue section was further rinsed with distilled water and dehydrated and cleared using xylene. Finally, the stained tissue sections were evaluated and the results were tabulated. The entire procedure is tabulated with concentration of each reagent in Fig. 1.

## 3 Results

The histological evaluation of developing rabbit tooth germ tissues stained with Modified Gallego stain revealed distinct and reproducible color differentiation among various tissue types, aiding in the identification and characterization of both mineralized and soft tissues.

**Fig. 2:**
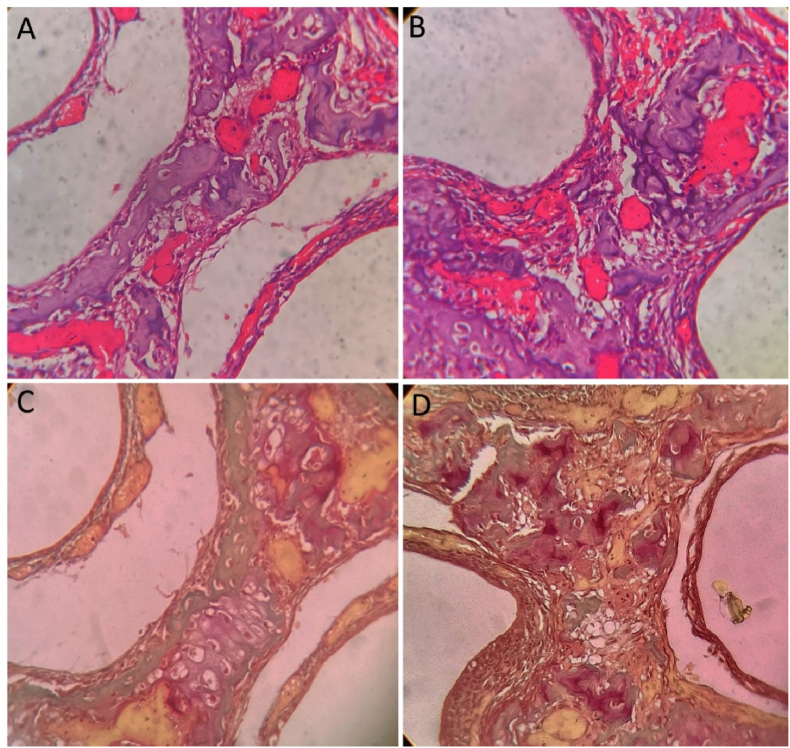
**Comparison of bone and chondroid tissue staining in H&E (A&B) and Modified Gallego-stained sections (C&D).** The chondroid bone is prominently stained bright pink, indicating its cartilaginous matrix composition and transitional nature between cartilage and bone. In contrast, adjacent regions of mature bone exhibit a green hue, consistent with mineralized bone matrix.

The chondroid tissue, observed in regions suggestive of endochondral ossification or transitional zones, exhibited a bright pink coloration, indicative of its unique extracellular matrix composition rich in cartilaginous proteins. In contrast, areas representing osteoid, the unmineralized pre-bone matrix, stained yellowish-green, distinguishing it from mature mineralized bone and allowing for the visualization of early bone formation (Fig. 2).

**Fig. 3:**
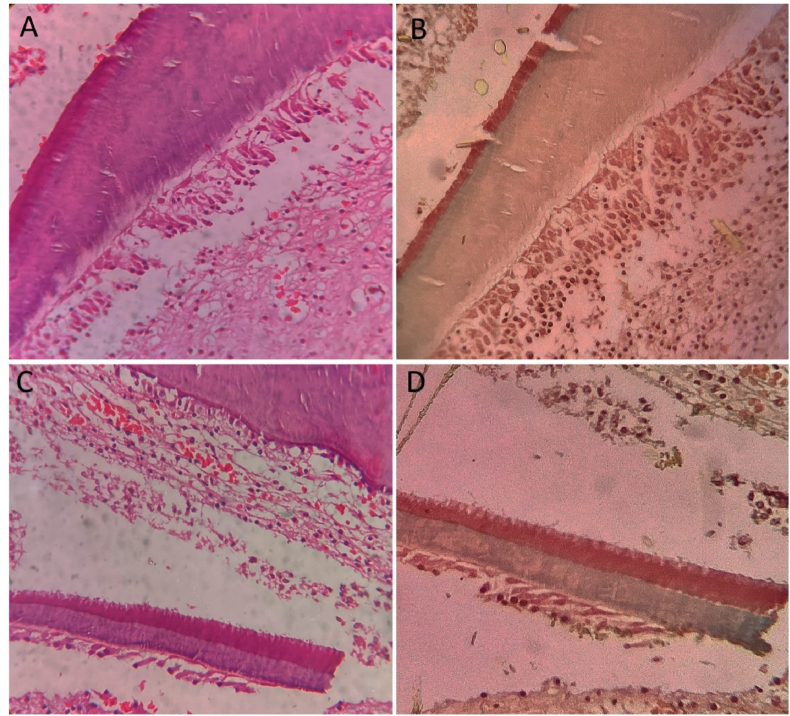
**Comparison of dental hard tissue staining in H&E (A&C) and Modified Gallego-stained sections (B&D).** Enamel appears red, dentin is stained green, and predentin displays a lighter green hue adjacent to the dentin layer.

Dentin and bone tissues both displayed a green hue, consistent with their high mineral content and similar collagenous matrix characteristics. Notably, predentin, the newly secreted and less mineralized precursor to dentin, displayed a lighter green shade, facilitating differentiation from mature dentin. This gradation offers insight into the progressive nature of mineralization during odontogenesis (Fig. 3). The enamel, observed in the crown region of the developing tooth germ, appeared red, differing from earlier reports describing it as pink, potentially due to stage-specific differences in mineral density or organic matrix composition. This distinct coloration underscores the potential of Modified Gallego stain to identify subtle variations in tissue maturation.

The connective tissue displayed a dichromatic staining pattern. The connective tissue within the dental papilla and surrounding areas of active bone formation stained pink, while the connective tissue located peripherally—surrounding the tooth germ and beneath the oral epithelium—stained green, suggesting regional variation in collagen content and organization.

**Fig. 4:**
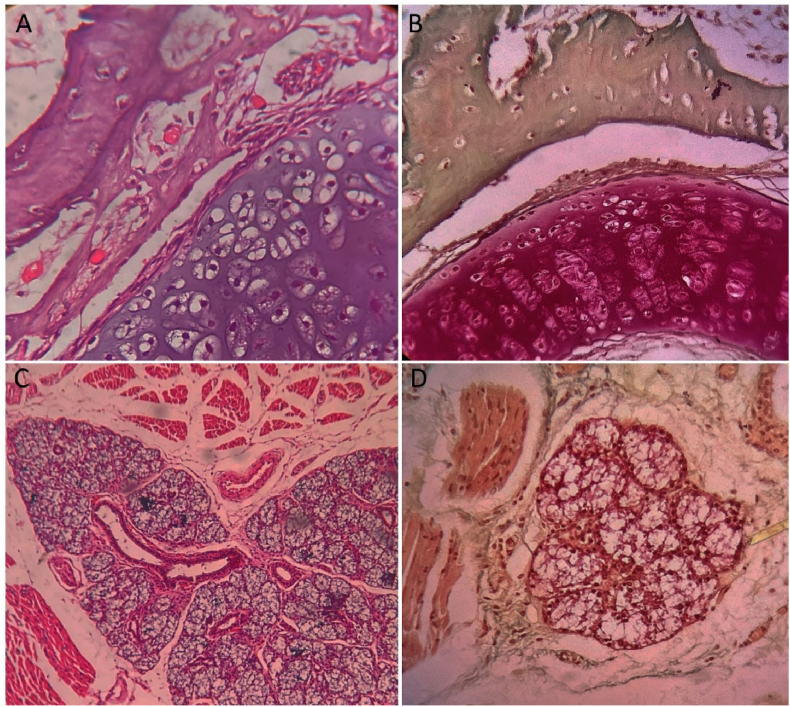
Differentiation of nasal cartilage, bone, and salivary gland acini using H&E (A&C) and Modified Gallego stain (B&D).

The nasal cartilage and the surrounding bone exhibited similar staining patterns to those observed in the maxillary region around the developing tooth germ. Additionally, salivary gland acini, visible in the adjacent soft tissue regions, stained a bright pink, likely due to their rich cytoplasmic content and glandular architecture. Skeletal muscle fibers, observed in proximity to the developing oral structures, exhibited a reddish-yellow coloration, which reflects the sarcoplasmic and connective components of the muscle (Fig. 4). Overall, the Modified Gallego stain provided excellent contrast and clarity in distinguishing between mineralized and soft tissues, as well as among tissues at various stages of development. These staining characteristics support its applicability in developmental histology and potentially in pathological tissue analysis.

The nasal cartilage, composed primarily of chondroid tissue, is stained a bright pink, reflecting its cartilaginous matrix rich in proteoglycans and collagen. In contrast, adjacent bone tissue displays a green hue & the salivary gland acini, located in the surrounding soft tissue, appear bright pink. These coloring patterns provide valuable insights into the composition and structure of the analyzed tissues, indicating the presence of distinct cellular components and extracellular matrix. From the literature search, the staining of various hard tissues with Modified Gallego is listed in Table 1.

**Table 1:** Summary of the staining patterns of different hard tissues using the Modified Gallego stain, as documented in the literature.

MODIFIED GALLEGO STAIN		
AUTHOR NAME & YEAR	TUMORS	INFERENCE
Satheesan E et al., 2016	Tooth germ	Pre dentin – Green
	Extracted teeth (Decalcified section)	Cementum – Red, Bone – Green
Mudhiraj PV et al., 2017	Extracted teeth (Ground Section)	Enamel – Pink, Dentin – Green, Cementum – Dark red
	Odontome	Dentin - Green, Cementum – Red
	CEOT	Liesegang rings – Green
	Osteoma	Bone – Green
	Osteomyelitis	
	Odontogenic fibro-myxoma	
Kunche A et al., 2017	POF	Bone – Green blue, Cementum – Red, Collagen – Blue
	OF	
	Odontoma	
	COF	
Afroze SN et al., 2018	Extracted teeth (Decalcified section)	Cementum – Red, Dentin & Bone – Green
	Odontome	Dentin/Bone-like material – Green, Cementum – Red
	CEOT	Amyloid like material – Pink
	Dentinogenic ghost cell tumor	Dentin/Bone-like material – Green
	Ameloblastic fibro dentinoma	
	Odontoameloblastoma	Dentin/Bone-like material – Green, Enamel-like material – Pink
	AOT	Enamel-like material – Pink
Dhouskar S et al., 2019	OF	Bone – Green
	COF	Bone – Green, Cementum – Red
Agrawal RS et al., 2020	FD	Cementum – Red Bone – Greenish yellow
	OF	
Krishnakumar M et al., 2024	Archegonous cystic odontoma	Dentinoid – Green, Bone – Green, Collagen – Green pink
	Dentinogenic ghost cell tumor	
	Calcifying Odontogenic cyst	
	Calcifying Odontogenic cyst	

While the study primarily focused on qualitative assessment of staining patterns using the Modified Gallego stain, a semi-quantitative approach was employed to systematically record and tabulate observations based on staining intensity and tissue differentiation. These parameters were graded and recorded across multiple sections to ensure consistency and reproducibility. All stained histological sections were evaluated independently by two calibrated observers, both of whom were blinded to the specimen groupings and experimental conditions to minimize bias. Before the assessment, inter-observer calibration was conducted using a subset of slides to ensure consistency in grading criteria. The observations were recorded using a semi-quantitative scoring system for staining intensity, where 0 represented no staining, 1 indicated weak staining, 2 denoted moderate staining, and 3 signified strong staining (Table 2). When the observers disagreed, a consensus was achieved through joint re-evaluation. This protocol was implemented to enhance the objectivity and reproducibility of the results.

**Table 2:** **Semi-Quantitative Assessment of Staining Patterns in Rabbit Tooth Germs Using Modified Gallego Stain** (∗Staining intensity scored on a 0–3 scale 0 = No staining, 1 = Weak, 2 = Moderate, 3 = Strong).

Tissue Type	Staining Color	Staining Intensity (Mean Score∗)	Frequency of Positive Staining (%)	Observations Summary
Dentin	Green	2.5 ± 0.4	90 %	Demarcated, uniform staining
Enamel	Red	3.0 ± 0.0	100 %	Intense, sharp contrast with adjacent tissues
Bone	Green	2.8 ± 0.6	90 %	Slight overlap with cartilage in few areas, distinguishable margins
Cartilage	Bright Pink	2.9 ± 0.5	95 %	Demarcated, Uniform staining

## 4 Discussion

Odontogenesis means the formation of a tooth & its surrounding structures. The formation of hard tissues has an organised & sequential pattern with dentin forming first followed by induction of inner enamel epithelial cells to convert into ameloblasts & form enamel. Special stains are used in histopathology to identify the tissues as they stain with different colours due to variations in permeability & molecular size. Special stains were mostly used in pathological conditions to identify the nature of tissues.^6^^,^^7^ We have used special stains to highlight the pattern of mineralization of the forming hard tissues such as enamel, dentin & bone. There were only a few articles regarding special stains for the calcified structures during odontogenesis in the literature. In our study, the enamel during odontogenesis appeared red in Modified Gallego stain, whereas the previous studies mentioned that the enamel is stained pink.^8^^,^^9^ This color variation could be attributed to differences in enamel maturation stages or species-specific variations in enamel matrix composition during development. It also raises questions about how stain uptake differs based on the mineral content and protein structure of early enamel. Cementum appeared red to bright red; however, cementum has yet to develop in our tissues taken for the study.^10^, ^11^, ^12^

Dentin, a crucial marker of early mineralization, stained green in our specimens, consistent with findings from previous studies in fully developed teeth and in odontogenic pathologies such as odontomas and calcifying epithelial odontogenic tumors.^5^^,^^13^, ^14^, ^15^ Notably, we observed a gradation in staining: predentin exhibited a lighter green shade compared to mature dentin, a differentiation not previously reported. This contrast may reflect the lower mineral content in predentin and highlights the potential of Modified Gallego stain in distinguishing early matrix from fully mineralized dentin during tooth development.

The bone formation surrounding the tooth germ, derived from the dental follicle, was also distinctly stained. The osteoid appeared green, while areas of chondroid bone stained pink, indicating differing matrix compositions and possibly distinct ossification pathways. These findings align partially with prior literature, which describes bone in pathological conditions (e.g., ossifying fibroma, osteoma, and osteomyelitis) as appearing greenish to bluish under Modified Gallego staining.^16^, ^17^, ^18^ However, our study demonstrates that bone undergoing intramembranous ossification in a developmental setting can show similar chromatic responses, reaffirming the stain's relevance beyond disease contexts.

Finally, the collagen fibers showed a dichromatic pattern—a novel observation in contrast to previous reports.^19^^,^^20^ In our study, collagen fibers near active bone formation and within the tooth germ appeared pink, while those surrounding the tooth germ and adjacent epithelium stained green. This variation could reflect changes in collagen density, orientation, or degree of mineral association, and underscores the sensitivity of Modified Gallego stain to subtle extracellular matrix differences during development.

This study unraveled that there was a difference in the staining pattern in the hard tissues during development, after its completion & in pathological conditions. In clinical practice, this enhanced tissue differentiation can aid pathologists and dental specialists in more accurately diagnosing developmental anomalies, fibro-osseous lesions, and other conditions involving abnormal mineralization, especially where conventional stains fall short. By providing clearer histological distinction, especially at early stages of tissue formation, this staining technique could support earlier detection and better characterization of odontogenic tumors, dysplasias, or cemento-osseous pathologies. Modified Gallego stain may allow for the identification of early-stage hard tissue formation within tumors, offering insight into the mineralization process before it fully matures. This capability enables comparisons between early and mature stages of tumor calcification, which may improve diagnostic accuracy and help in staging or prognostic evaluation. Additionally, the stain's usefulness in assessing tissue integration makes it relevant for monitoring regenerative procedures or biomaterial performance. Overall, the Modified Gallego stain holds promise as a diagnostic and research tool in both routine pathology and advanced dental investigations.

## 5 Limitation

Though the study emphasises the hard tissue development and maturation, it has its limitations. The sample size was limited which may not fully represent the broader developmental variations across different stages. The interpretation remained semi-quantitative, despite efforts to minimize bias through observer blinding and calibration. Future studies should aim to include a larger and more diverse sample set across various developmental stages and species to validate the generalizability of the Modified Gallego stain in odontogenesis. Comparative analyses with other special stains, such as Von Kossa or Alizarin Red, would help establish its relative sensitivity and specificity in differentiating mineralized tissues. Additionally, integrating advanced techniques such as immunohistochemistry, energy-dispersive X-ray spectroscopy (EDX), or micro-CT imaging could provide complementary molecular or structural insights, enhancing the interpretation of staining outcomes. Standardizing the staining protocol for developmental studies and evaluating inter-laboratory reproducibility would further strengthen its application in both research and diagnostic settings.

## 6 Conclusion

The findings of this study highlight the potential application of Modified Gallego stain in diagnosing and understanding dental pathologies at early stages of development. Further research and validation of these findings could lead to the wider adoption of Modified Gallego stain in dental histopathology, offering new insights and diagnostic capabilities in the field of oral pathology.

## Patient’s consent

Not Applicable.

## Authors contribution

Conceptualization: Suganya Panneer Selvam.

Methodology: Shweta Mary Reddy & Sandhya Sundar.

Writing – Original Draft – Shweta Mary Reddy & Suganya Panneer Selvam.

Writing – Review and Editing – Sandhya Sundar.

Visualization - Suganya Panneer Selvam & Ramya Ramadoss.

Validation - Ramya Ramadoss.

## Ethical clearance

The study protocol was duly approved by the institutional ethical committee (BRULAC/SDCH/SIMATS/IAEC/04–2022/105).

## Source of funding

Nil.

## Declaration of competing interest

The authors declare that they have no known competing financial interests or personal relationships that could have appeared to influence the work reported in this paper.
